# Role of Moderators on Engagement of Adolescents With Depression or Anxiety in a Social Media Intervention: Content Analysis of Web-Based Interactions

**DOI:** 10.2196/13467

**Published:** 2019-09-26

**Authors:** Carolyn Windler, Maeve Clair, Cassandra Long, Leah Boyle, Ana Radovic

**Affiliations:** 1 University of Pittsburgh School of Medicine Pittsburgh, PA United States; 2 University of Pittsburgh Pittsburgh, PA United States; 3 UPMC Children’s Hospital Pittsburgh, PA United States; 4 Chatham University Pittsburgh, PA United States; 5 Division of Adolescent and Young Adult Medicine University of Pittsburgh School of Medicine UPMC Children's Hospital of Pittsburgh Pittsburgh, PA United States

**Keywords:** moderator, social media, engagement, adolescents, mental health

## Abstract

**Background:**

The Supporting Our Valued Adolescents (SOVA) intervention aims to use a moderated social media website to encourage peer discussion about negative health beliefs, which may prevent treatment uptake. Web moderators with a background in behavioral health are used to facilitate peer conversation to promote a sense of community, provide social support, and ensure safety.

**Objective:**

Although moderation is a core component of this intervention, little is known on best practices for moderators to ensure safety while encouraging engagement. This study sought to describe interactions between moderators and peer users and understand moderator experiences through individual interviews.

**Methods:**

Adolescents and young adults aged 14 to 26 years with depression or anxiety history were recruited for a usability study of the SOVA intervention. During this study, 14 moderators were trained to regularly review comments to blog posts for safety, facilitate conversation, and correct misinformation. A total of 110 blog posts and their associated comments were extracted and coded using a codebook based on items from the supportive accountability model and a peer social support analysis. Closing interviews with 12 moderators assessing their experience of moderating were conducted, recorded, and transcribed. Blog post text and comments as well as transcripts of moderator interviews were assessed using a thematic analysis approach, and blog posts were examined for trends in content of moderator comments comparing blog posts with differences in comment contributor order.

**Results:**

There were no safety concerns during the study, and moderators only intervened to remove identifiable information. Web moderators exhibited elements of supportive accountability (such as being perceived as experts and using verbal rewards as well as offering informational and emotional support). When the moderators provided the last comment under a blog post, thereby potentially ending contribution by users, they were at times found to be commenting about their own experiences. Moderators interviewed after completing their role expressed challenges in engaging users. A cohort of moderators who received more extensive training on supportive accountability and peer social support felt their ability to engage users improved because of the training.

**Conclusions:**

Moderators of a Web-based support site for adolescents with depression or anxiety were able to ensure safety while promoting user engagement. Moderators can elicit user engagement by offering gratitude and encouragement to users, asking users follow-up questions, and limiting their own opinions and experiences when responding to comments.

## Introduction

Web-based interventions enhanced with social media components offer a novel approach to intervening on key mechanisms which may increase adolescent use of mental health services. These key mechanisms include addressing parents’ and adolescents’ health beliefs [1,2], offering emotional support [3,4], and facilitating communication about mental health with other adolescents and other parents. There has been a generational shift regarding the approach to medicine, such that patients consult Web materials before visiting a physician in person [5]. Furthermore, adolescents with depression often use social media to discuss their mental health beliefs to obtain emotional support from peers [6]. Although research shows that depressed adolescents may benefit from talking to others on the Web about their mood [7], they can also encounter negative content and feedback from others, including cyberbullying [6,8]. Website interventions employing social media components therefore should be moderated to first and foremost ensure safety and encourage positive Web-based interactions between users [9]. Moderators have been used successfully to protect users [10], and moderators who exhibit skills and knowledge can promote engagement in Web-based social support groups [11].

Social media allows interventions to target individuals who may otherwise be hard to reach, with moderators as a means to reduce risk [12]. For example, those who are shy/quiet may be more likely to share their feelings on the Web [13]. Several studies have demonstrated the success of using social media as an intervention for depressed young people [7], using moderation to limit adverse events [14]. According to Kraut and Resnick’s book *Building Successful Online Communities: Evidence-Based Social Design*, moderation of Web-based content is particularly important in ensuring safety and blocking inappropriate messages [15]. Furthermore, users are more satisfied with the moderator if screening decisions are fair, criteria are clear/consistent, and moderation is provided by impartial members of the community. The supportive accountability model, created by David Mohr, provides a structured framework for Web-based coaches to simultaneously support and hold users accountable when actively engaging in electronic health (eHealth) behavior change interventions [16]. Mohr and Kraut’s work dictates that users are more likely to adhere to treatment when users view coaches as individuals who are benevolent and trustworthy and as having expertise. Coaches should provide user-centered benefits, be specific about expectations, involve the user in making decisions, tie outcomes to a user’s larger life goals, focus on process not outcome, and always justify actions. Mohr warns against external rewards, such as money, which may undermine intrinsic motivation and limit long-term success. In contrast, verbal rewards such as positive feedback are encouraged as a means to enhance intrinsic motivation. The supportive accountability model also considers mimicking the communication style of users in content, tense, and tone, known as *paralinguistic mirroring*, as useful for increasing comfort and trust between users and coaches.

The Supporting Our Valued Adolescents (SOVA) social media website is a Web-based intervention for adolescents and young adults aged 14 to 26 years with depression or anxiety. The site uses daily blog posts to address mental health and increase the perceived need [17] for services with the goal of creating a Web-based community which mainly has anonymous discourse via comments to blog posts [18]. Stakeholder feedback and established user-centered methods informed the design of SOVA, with subsequent usability testing showing user-friendliness and no safety concerns, with high importance placed by users on the need for website moderation [19]. The greatest concern for stakeholders in using social media for suicide prevention was that the moderators would not have sufficient training to be able to intervene in a safety situation [20]. Thus, one of the key aspects in SOVA site development was ensuring round-the-clock site moderation by behavioral health professionals or trainees. Given that anonymity was important to stakeholders, the moderation was crucial to maintain trustworthy content. One of the challenges surrounding the SOVA project involved identifying strategies a moderator can use to strike an equilibrium between the dichotomy of a disciplinary, and possibly intrusive, role (ensuring user safety and accurately shared information), with a role to build a Web-based community (facilitating conversation and user engagement). This study explored the moderator process itself and how moderators can balance these seemingly opposing roles.

Few previous studies assessed how Web moderators can engage users, especially in adolescent internet support groups. As a part of a usability study of the SOVA intervention [18], we had an additional opportunity to examine the role of the Web moderator through observational (comments) and self-report (interview) data. This study sought to understand the role of the moderator more clearly through 2 specific aims: (1) to describe the extent to which moderator interaction ensures safety, facilitates or deters peer conversations, exhibits principals of supportive accountability, and provides social support (and types provided); and (2) to understand and describe the experience and feedback from moderators on the SOVA website to inform future changes to moderator training.

## Methods

### Sample

Adolescents and young adults aged 14 to 26 years with a self-report of experiencing symptoms of depression or anxiety were recruited for a usability study of the SOVA intervention which is described in detail in a previous paper [18]. Briefly, during that study, users who logged on to the website were able to comment on daily blog posts, all of which were written by moderators at that time, except for 2 written by one user who was piloting peer blogging. Of the 96 participants recruited, 41 users ever logged in, and only 16 users ever commented, with 5 users commenting more than 5 times.

During the usability study, 14 moderators were involved in site moderation with up to 4 at a time, always including the principal investigator (PI) as a backup. Moderators included research assistants who were also licensed social workers, graduate students in social work, graduate medical students in psychiatry, or graduate students in psychology. The PI was not interviewed for this study.

### Procedure

Moderation occurred through receiving mobile phone notifications for a research study email, which would forward new comments posted on a SOVA blog post. Moderators worked in shifts, and during a shift, they were vigilant to check for new emails at least every 3 hours. Any time that was not actively moderated by these individuals was moderated by the PI.

### Moderator Training

All moderators received a 2-hour live training by the PI with specific case examples describing potential moderating scenarios. The PI was also made available for questions throughout training and while moderating. Both Mohr and Kraut’s work investigating the role of Web moderation informed the training for moderators on the SOVA website and were subsequently used to evaluate the success of this moderating role [15,16]. This training covered background on the problem of inadequate treatment of depression and anxiety; the design, pilot work, and content of the SOVA intervention; the supportive accountability model (incorporated for last half of moderator trainees) that describes a framework for what characteristics may be desirable in the Web coaches to increase behavioral technology engagement [16]; assessing suicidality (eg, what are thoughts, plan, intent); and reviewing how to react to a crisis or emergency situation (eg, a user posts suicidal content).

Moderation of the SOVA website involved screening all blog post comments within 3 hours after they were published and judging whether to respond. Moderators were instructed to review and respond to comments as necessary to (1) facilitate conversation and indicate to users that someone read their comment, but only if no other user had commented after 24 hours to not stifle conversation; (2) correct misinformation (eg, regarding incorrect medical advice); (3) remove identifiable information; (4) address cyberbullying; (5) be available to give feedback and advice; and (6) screen for safety. Users were provided with ground rules of SOVA site use (Multimedia Appendix 1) which moderators were asked to enforce.

### Information Collection and Analysis

#### Blog Post Comment Extraction and Coding Process

The blog post and their associated comments were extracted from the website database for 110 blog posts from April 2015 to February 2017 and downloaded into NVivo (QSR International) qualitative software. A codebook was developed which included labelling of comment author (user vs moderator) and author order, and individual codes based on items retrieved from the supportive accountability model [16] and peer social support analysis [21] (Multimedia Appendix 2). Although user comments were coded for social support provided to other users, the analysis focused on moderator comments and moderator-user interactions.

The first 50 blog posts were coded by 2 coders independently using an initial codebook. They then met with the PI to review codes and modified the codebook based on this feedback, mostly regarding the definition of specific codes. The units of coding used were one entire comment; although at times, parts of a blog post could be included as a *comment* if it included codes perceived to be the blog post writer talking to the community. For example, moderators would often include questions at the end of a blog post for the community. Individual comments could be coded with multiple codes if fitting under multiple categories. The modified codebook was used by 2 coders to recode the initial 50 blog posts until a percent agreement score of 90% was achieved (our criterion was at least 80%, there were 3 iterations of coding), after which 1 coder coded the rest of the blog posts. Mismatches were adjudicated by the PI. The assumption was made that the user who sees an article written by the moderator also perceives this as an interaction with the moderator when commenting on that post. Blog posts were then broken up into categories based on the order of individuals commenting (eg, user, moderator, and user). Blog posts were separated into categories for which conversation continued after moderator response and those for which conversation ended after moderator response. Codes within these categories were compared for overlying themes.

The analysis of comments was approved by the University of Pittsburgh Human Research Protection Office (Institutional Review Board [IRB]).

#### Moderator Interviews and Coding Process

As moderators discontinued their work with SOVA, usually because of a finishing graduate school practicum or job transition, they were asked to participate in a closing interview conducted by a research assistant. Individual interviews were conducted with 12 moderators, all of whom had moderated for a period of 6 months or more. Interview questions assessed the experience of moderating and perceptions about training. These were recorded, transcribed, and double coded until greater than 80% agreement was achieved across all nodes. Interviews were transcribed verbatim excluding filler words (eg, like and um). A prespecified codebook (Multimedia Appendix 3), updated during coding, was utilized. This interview study was found to be exempt from IRB approval.

### Analysis

We used a template analysis approach for both analysis of the blog post comments as well as the moderator interviews, using a prespecified codebook and a hierarchical approach to the coding, being open to future changes in codes as coding progressed [22]. For blog post comments, an exploratory analysis was conducted to assess differences between the content of comments for when conversation stopped after the moderator commented versus when it continued. Researchers examined the blog posts from this final category where both moderator and user responded and explored trends within them to ascertain if moderator involvement or the order of involvement seemed related to the amount of further user comment contributions as a proxy for engagement. For moderator interviews, thematic content analysis was conducted to understand overarching themes of moderator experiences and opinions. Findings were discussed with previous moderators as a method of member checking [23].

### Ethical Approval

This work was approved by the IRB of the University of Pittsburgh (PRO15060158).

## Results

### Blog Post Comment Analysis

Out of 363 total blog posts, the 110 that received comments (2 of which were written by a user) were coded and assessed. First, there were no safety concerns throughout moderating. There were fewer than 5 instances that moderators intervened to remove identifiable information from comments or correct misinformation. There were no instances of cyberbullying or crisis situations. Moderators most often commented to facilitate conversation or offer feedback and advice.

Of the 110 blog posts, there were 8.1% (9/110) where only the moderator responded; 44.5% (49/110) where only one or more users responded; and 47.2% (52/110) where both the moderator and user(s) responded. Of the 52 blog posts that received user and moderator comments, for 36% (19/52; 1 user commenting) and 6% (3/52; multiple users commenting) the conversation stopped after the moderator responded (moderator stop). For 58% (30/52) the conversation continued after the moderator responded (moderator continues).

In blog posts where conversation stopped, moderators were found to be self-disclosing, commenting about their own experiences. Alternatively, the conversations that continued (eg, user, moderator, and user) revealed several trends. These trends included the moderator utilizing emotional support including thanking the user, asking them a question, asking for their tips, and offering them encouragement. As further detailed below, moderators commonly displayed elements of Mohr’s supportive accountability model in conversations which continued after moderator input by (1) sharing or displaying moderator expertise, (2) verbally rewarding user, and (3) mirroring (eg, mimicking the same emoticon as a user) [16].

**Figure 1:**
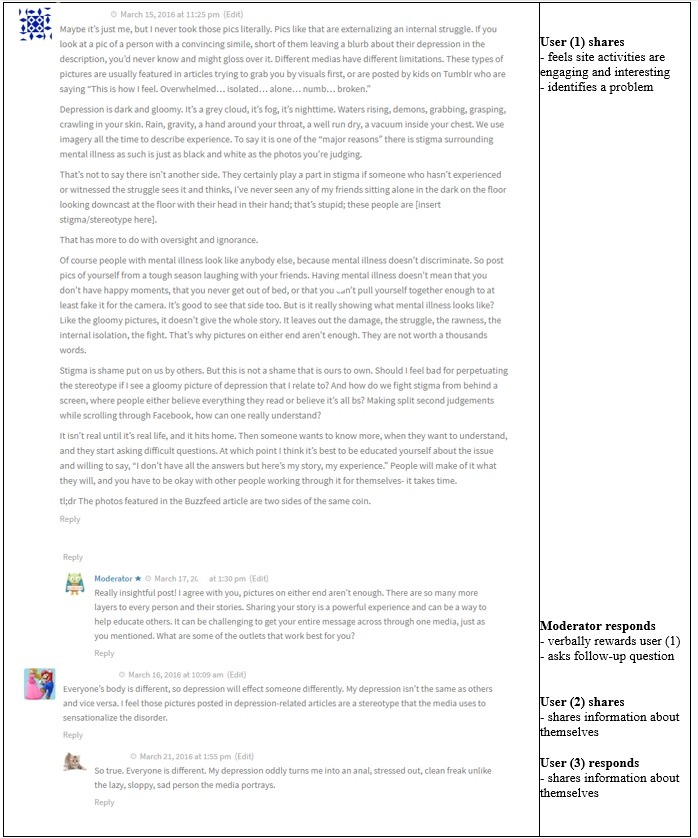
Moderator continues conversation through rewards and questions.

An example of a blog post where the conversation continued after the moderator responded was one called, “What Depression Really Looks Like,” which addressed the stereotype that people who are depressed will look depressed, when often that is not the case. The question asked at the end of the blog post was, “Do you think the pictures used to portray depression play a role in the stigma around it?” A user responded after which the moderator responded, and a conversation followed (Figure 1). In this case, the moderator asked a follow-up question. They also verbally rewarded the user, which was one of Mohr’s motivational constructs for increasing engagement and motivation.

**Figure 2:**
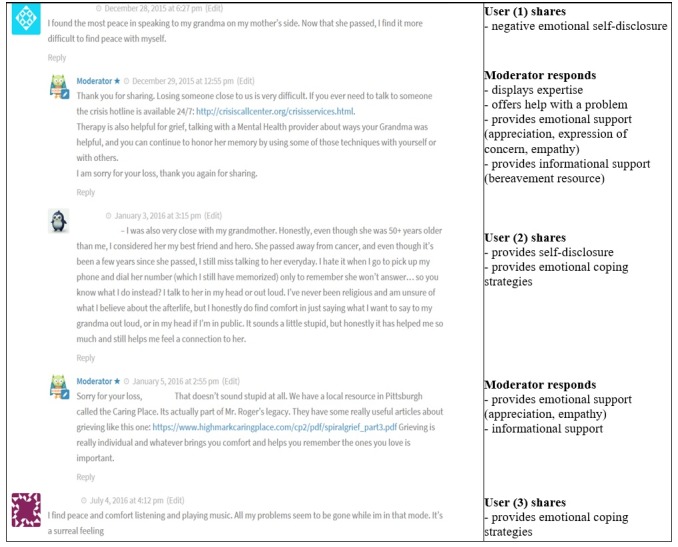
Moderator continues conversation through expertise and support.

Another example of continued conversation addressed negative emotions, specifically, the loss of a loved one. In this case, a user commented, a moderator responded, and a conversation continued (Figure 2). Here, the moderator displayed expertise and a potential solution to the user’s problem. The moderator also provided support, both emotionally and in the form of informational resources.

**Figure 3:**
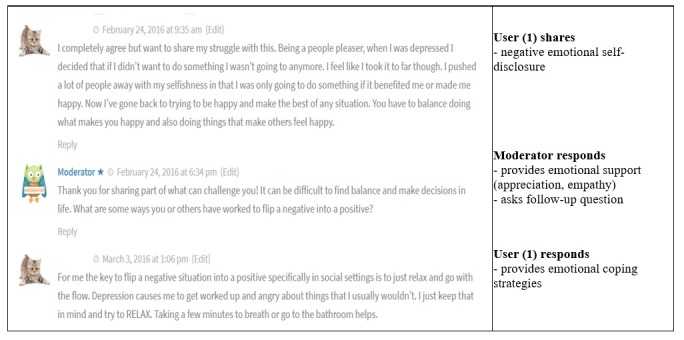
Moderator continues conversation through support and question.

A final example of continued conversation was one surrounding a post that discussed depression and how it manifests in the individual. The same user commented back to the moderator in this case (Figure 3). The moderator first provided emotional support by thanking the user for their post and then asked a follow-up question. The same user responded to the moderator’s post by elaborating on the situation and discussing relaxation techniques.

**Figure 4:**
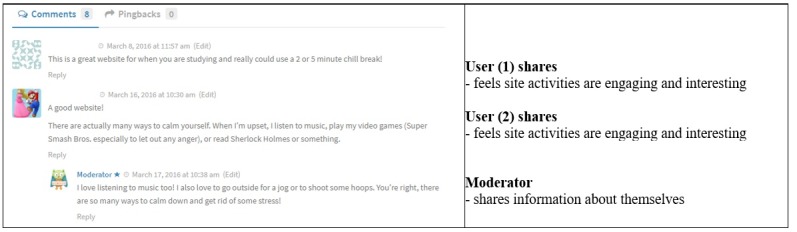
Moderator stops conversation.

An example of a blog post where the moderator stopped the conversation was one called *Calm.com*, which provided information about a relaxation website. The question asked at the end of the article was, “Do you have the calm app? What do you think of it? What do you think of having something that allows you to put away everything else for just a few minutes to have some time for just yourself? Tell us about it in the comments!” A user responded after which the moderator responded, and there was no further conversation (Figure 4). In this example, the moderator was commenting on their own experiences using positive self-disclosure, and the conversation ceased to continue.

In the first example, the moderator’s question was not answered by the original user but did not prevent further commenting. In the second example, the moderator provided both emotional and informational support, and neither comment deterred further user interaction. In the third example, the moderator asked the user a question, to which the same user responded, thus fostering further engagement. In the final example where the conversation stopped, perhaps the moderator commenting about their own experiences may have deterred further user response.

An analysis of the coded comments revealed several trends in the way moderators engaged on SOVA. All codes occurring more than 10 times in blogpost comments can be found in Multimedia Appendix 4. In 22 blog post comments, moderators provided expertise in the form of either recommending local resources such as crisis centers or education material or sharing professional knowledge on a variety of topics related to mental health or use of social media. In 3 comments, users perceived expertise from moderators and responded with acknowledgement and appreciation, including statements such as “these are really helpful.” For example, in response to a quote about friendship, a user replied, “this happened to be just the right time that I needed to hear a quote like this. Thank you.” In 14 comments, the moderator responded to users’ comments by asking follow-up questions such as “Was it as difficult for you to share with those you trust? Do you or anyone else have any tips on how to approach the subject?” to try to increase user interest and engagement. Out of these 14 comments, 11 resulted in a user responding to the question asked by the moderator, whereas 3 did not. However, those 3 that did not incite a user response to the posed question did not limit different users from commenting further within the same thread.

Moderators also offered potential solutions to problems users expressed in 10 comments, often suggesting ways to avoid rumination and adopt more positive attitudes. An example of a moderator providing a solution was when giving advice to someone struggling with sleep, encouraging the user to practice “controlling your exposure to light, creating bedtime rituals that help with relaxation, and keeping the bedroom cool and quiet.” Users expressed that they learned something new from the site on 10 occasions, though not overlapping with moderators’ solutions but in response to informational posts or tips from other users. In 51 comments, moderators provided users with verbal rewards including phrases such as “thanks for sharing!” and “great point!”

Users occasionally shared desire for emotional support or information (2 and 4 comments, respectively). Of these 6 cases, the moderator responded 5 times, and another user responded once. For example, when a user asked, “How would the average person know when someone’s having a panic attack?” the moderator responded with details and a link to a website outlining mental health first aid guidelines. Moreover, moderators responded thoroughly to all aspects of the users’ comments—thanking them for sharing, addressing the story they shared with positive affirmations, answering any questions they may have posed, and occasionally sharing some new information or asking a follow-up question to promote discussion. For example, when a user stated, “I have a hard time wording things,” the moderator asked, “What are some ways you might try to word what you want to say from reading this article?” which prompted the user to continue the conversation. This demonstrates the moderator reacting to a disclosure by the user and using an open-ended question to further discussion.

Emotional support was provided most commonly by users, but also by moderators, for a total of 57 times. This support was most often in the form of moderators’ or users’ acknowledgment of what the original user posted (“I agree, journaling can be a great way to deal with things”), or from users sharing similar stories of one’s own, saying things such as “I completely agree [and I, too] want to share my struggle with this.” Informational support was also provided by both users and moderators 167 times. This involved sharing resources and strategies with fellow users, including providing emotional coping strategies, such as links to websites as mentioned or advice on how to deal with therapists, physicians, stressful situations, or negative thoughts.

In 63 comments, users made positive remarks highlighting the value of the SOVA website, most often responding to blog posts, but also to other users’ comments or moderator’s comments, such as “This was very insightful and helpful” and “I’m definitely going to try [that].”

### Moderator Interview Analysis

Moderators shared challenges in engaging users in thoughtful conversation during the usability testing phase. They believed this was because of a limited number of users on the site at one time, and iterative updates to website functionality (eg, new article notifications not working). They also shared challenges engaging users because of the nature of users themselves, “I think it just takes a unique type of adolescent to want to engage online with this type of site...it really makes sense that our adolescents do use a lot of online communities and that this is something they would be interested in, but I think it takes a lot of forethought and insight that adolescents might not have about their own mental health.” One moderator mentioned some of the challenging aspects surrounding engaging users, “How do you create a conversation...if you are responding to a comment and you say, ‘...That’s a really good point...’ how do you make it into a conversation so that they want to respond more? Or how do you get other users to interact with each other? That is an important role of the moderator to create that space where conversations can happen.” Following concerns from moderators that more guidance on peer engagement and crisis training were needed, this was updated in training procedures, and later moderators perceived that training was adequate and valuable to performing their duties, especially when previous or existing moderators were accessible and approachable. Often, it took the moderators several weeks to become fully comfortable with their duties and those with a stronger background in mental health reported feeling more prepared. They felt their role in commenting was relatively unimportant when compared with keeping the site and users safe. Taking that into account, there were no safety concerns, and moderators found using a research study mobile phone with all new site content emailed to the mobile phone a feasible way to incorporate moderating with balancing their other daily tasks, so they could be available 24×7. Overall, the moderators stated that they most enjoyed interacting with other users (including replying to comments), gaining mental health experience and training, and feeling as though they were making a difference through their role. The moderators had many positive things to say about the study, “I like that the role [of the moderator] is important, even though I do not necessarily always have a lot of interactions with the users. I know that the subscribers know that someone is there to make sure the information is accurate and that it is a safe environment to discuss and talk is good. I like knowing my role is needed.”

## Discussion

### Principal Findings

In this study, after examining the role of the moderator in a Web-based intervention for adolescents with depression or anxiety, we found that moderating such an intervention was feasible and resulted in no safety concerns. Additionally, moderators exhibited various approaches that may impact user engagement. Moderators themselves expressed satisfaction with receiving training on techniques which may enhance user engagement and keep users safe on the Web, stating that they found the experience valuable. The findings of this study influenced changes to current moderator training including incorporating more feedback on emergency and safety protocols as well as enhancing feedback on how to increase user engagement by limiting moderator self-disclosure.

The role of the moderator in Web-based behavior interventions for adolescents and young adults is a necessary one to ensure the safety of users and quality of Web content. Moderation has been found to foster a welcoming and safe environment, prevent cyberbullying [24], and may even increase respect among users [11]. Moderation also improves quality and accuracy of information shared by users [11,25] and helps direct users to specific site content to address their unique needs [26]. In a study looking at YouTube TED Talk comments, moderators were mainly used to screen offensive content [27]. However, excessive moderation limited nonoffensive comments as well—demonstrating that it is important to be prudent when screening content to avoid limiting conversation. Along with promoting site safety and quality content, the moderating role in the context of our study involved facilitating Web-based peer-peer interactions, providing social support, and encouraging adolescents to communicate about mental health. This was achieved through follow-up questions and reliable responses to users’ comments within 24 hours. In our study and other eHealth interventions, periodic prompting by moderators can be an important tool to encourage participation [28]. Achieving this multiplicity of roles may at times be conflicting and complex, especially when attempting to convey a message through brief text. This challenge was often acknowledged and reflected upon in our moderator interviews. Nevertheless, in a variety of instances, moderators have been perceived positively with appreciation from users while serving the roles of encouraging user participation and filtering undesired content [29-31]. In the technological usability study of SOVA, we found no concerns from users regarding moderator interactions [18].

The supportive accountability model considers some moderator behaviors that may be exhibited in an interactive Web coaching scenario where traditional goal setting and rapport building may occur. As no such expectations were stated to users in our intervention, we did not expect to find some of the code families including bond, trustworthiness, benevolence, reciprocity, process expectations, and mirroring. Other code families from supportive accountability for which we did expect to find codes included: expertise, definition, identifying a problem, interest, reward, cues, social support, and seeking and providing emotional and informational support.

We found that varying moderator approaches may impact user engagement. Our findings led us to conclude that our concern of moderators deterring further conversation did not happen most of the time, but that at times when moderators exhibited self-disclosure, conversation from users would stop. Moderators may experience success by omitting their own opinions and experiences when responding to comments as this behavior may deter further discussion among users. One explanation for this finding is derived from the theory of Rogerian *client-centered* therapy, whereby person-centered dialogue is favored, such as encouraging patients to share or asking clarifying questions [32]. Working in this model, moderators would be expected to have more success when they avoid self-disclosures. Another explanation for this finding is that the user may perceive disregard from the moderator if the moderator shares a similar experience which they overcame. A study found that people who have experienced hardships appear less sensitive to those currently suffering and may be less likely to provide compassion [33]. The third possible explanation is derived from the concept of *acquiescence to authority*, whereby an uneven power dynamic between individuals may lead to acquiescence from the perceived person of lesser power when an authority figure makes a declarative statement [34]. In the context of this study, moderators are in a position of power as they oversee the website. Thus, the users may be less likely to respond to declarations, such as self-disclosures, from moderators and more likely to respond to moderators requesting feedback.

User engagement is essential for the success of Web-based interventions [15], which can be influenced by content, moderators, and users themselves. A study examining user engagement between different types of Facebook posts (eg, pictures, polls, multimedia, and questions) found that posts requiring a simple response, particularly polls, generated the greatest engagement [35]. Similarly, in our study, moderator-initiated posts that offered simple questions and moderator follow-up comments did incite a response from users, suggesting that users felt comfortable engaging with content sourced directly from the moderator.

We found that moderators exhibited techniques aligned with supportive accountability and social support and that users did seem to engage when these techniques were used, for example, when moderators would offer encouragement, informational or emotional support, display expertise, or offer verbal rewards. Despite this information on moderator strategies, the way users interact on the Web is, not surprisingly, user-dependent and varies significantly from person to person [27,36]. A study examining ratings of fictitious political candidates revealed that internal perception of self-efficacy plays a large role in how people are influenced by others on social media [37]. Stronger internal self-efficacy has been demonstrated to be a more powerful predictor of user response than the opinions of others, and individuals with depression and anxiety may have lower self-efficacy. This suggests that some users may be more susceptible to moderator influence than others. Moderators may also find success in altering their conversation style to directly target individuals, such as by mirroring the way in which the users they engage (ie, use of emoticons) [16]. Achieving this multiplicity of roles may at times be conflicting and complex, especially when attempting to convey a message through brief text.

Many therapy-based Web interventions reveal several important aspects of how humans may provide support. First, supported interventions, either by a therapist or nonhealth professional administrator, are more successful than unsupported mechanisms even when human resources are low [38]. Additionally, the method of support offered is not as important as consistency in meeting expectations and needs [39]. When Web-based therapists exhibit flexibility with guideline adherence and tailor support to individual client needs, adherence improves [40,41]. This challenge was also acknowledged and reflected upon in our moderator interviews. In some Web-based interventions, peer moderators acted as a bridge between adolescents using an intervention and expert moderators [24,26]. These peer moderators were often used to welcome new users and helped facilitate user engagement. Data suggest that peers can provide quality support with minimal training [42]. In a study utilizing peer support and assessing a social media intervention to prevent relapse in youth depression, moderation was found to foster a welcoming and safe environment and prevent cyberbullying [24]. Thus, a future improvement to moderation of the SOVA website could be made to include experienced fellow users as peer moderators.

This study has several limitations. First, the study was not designed to empirically examine the impact of utilizing specific techniques to increase user engagement. The data presented here are highly explorative and observational in nature, and a future experimental study would be needed to confirm our results, as we only describe trends in coding for when conversation continues and conversation stops. Supplementary analysis of moderator-user order on other social media platforms could be used to further investigate the trends found in this study. Thus, more qualitative research regarding moderator techniques in other behavioral interventions could further inform intervention design. In addition, the study was conducted during a feasibility and usability study with a smaller sample of users engaged on the site. If there were a larger sample of active users, there may be additional findings not accounted for in this study. Regardless, initial stakeholder feedback for the design of SOVA raised many concerns about safety [19], and we have been pleased that through social norm setting, we believe moderators have demonstrated the goal and purpose of the site and that this has limited negative interactions and any safety concerns. The role of a moderator involves delicate balance, as too much moderation can be perceived as surveillance and become detrimental to the success of the intervention [43]. Further research could assess the effect of moderators sending private messages to users in response to blog post comments, as an alternative to publicly responding back, and whether this promotes conversation among users. Feedback about moderator techniques such as this can have far-reaching effects for effectively increasing user engagement in potentially valuable social media interventions.

### Conclusions

The high rate of suicide and low rate of mental health treatment among adolescents highlight the need for social media interventions such as SOVA. Moderation is key for this sort of intervention to be both effective and safe. Moderators on the SOVA site elicited user engagement by offering gratitude and encouragement to users, asking users follow-up questions and limiting their own opinions and experiences when responding to comments. Users commenting on SOVA perceived that it had positive effects on increasing their adoption of healthy attitudes and behaviors. The research described is innovative specifically in investigating strategies a moderator can use to balance a potentially punitive and interfering role (enforcing site rules) with a supportive role (providing social support and facilitating conversation to promote peer-peer social support) for an adolescent Web-based support group intervention, all while effectively promoting user engagement.

## Appendix

Multimedia Appendix 1 User ground rules for Supporting Our Valued Adolescents website.

Multimedia Appendix 2 Blog post codebook and number comments coded.

Multimedia Appendix 3 Moderator interview codebook.

Multimedia Appendix 4 Codes occurring more than 10 times in blog post comments.

## Abbreviations

eHealth: electronic health
IRB: Institutional Review Board
PI: principal investigator
SOVA: supporting our valued adolescents
