# Supplementary material for: Role of Moderators on Engagement of Adolescents With Depression or Anxiety in a Social Media Intervention: Content Analysis of Web-Based Interactions
Source: JMIR Ment Health. 2019 Sep 26;6(9):e13467. doi: 10.2196/13467 (PMC6787534; doi:10.2196/13467)
Supplement: Multimedia Appendix 1 [file mental_v6i9e13467_app1.pdf]

Appendix 1: User Ground Rules for Supporting Our Valued Adolescents Website

COMMUNITY GUIDE

The SOVA website is a private online community and education website which is first being tried out in a research study. We want this to be a useful experience for you but we do ask you to follow a few rules to make this a good experience for everyone. By logging onto this website and participating, you agree to the terms below:

1. This site is moderated by professionals in behavioral health on a frequent basis, but we may not see comments immediately. This site is not for crisis services. If you or your child have a psychiatric emergency, please contact one of the resources on our crisis page or call 911.

2. We want everyone to feel comfortable saying what is on their mind. For this reason, we chose to make the site anonymous. That means every user will have an avatar, not a real picture of themselves, and they will have a screen name, and not use their real name.

Please do not share any information which can identify you like: your name, where you live, your contact information, or other personal details.

For example,

It is ok to say: I'm an eighth grader right now and there is definitely a lot of bullying at my school.

It is NOT ok to say: I'm an eighth grader at Shaler Middle School and there is definitely a lot of bullying at my school.

3. Do not ask or provide information to text, call, or meet with users outside of using this website.

4. This site is currently part of a research study. Please do not share your password or user ID with anyone else.

5. No bullying – If you don't agree with what someone else says it is ok to say your opinion, but please be kind and respectful when you do.

For example if someone says: "I am so mad that One Direction is popular – I don't like them at all."

It is OK to say: "I think they do a really good job. Check out this interview with the band – you might change your mind: (link)."

It is NOT OK to say: "Wow, you really don't have a clue about music. They are the greatest."

If you do see bullying, please send a private message to the therapist moderator to let them know.

6. Try not to type in ALL CAPS which can feel like you are shouting.

7. If you feel like you are getting very upset reading anything on the site, TAKE A BREAK. Sometimes when we are in a bad mood, we can get emotional easily. That is usually NOT a good time to read things which can make us more upset.

8. We think the links on the site are useful but we can’t vouch for their accuracy and can’t be held responsible for the content on the site or how private they are.

9. Its important for you to know that if you post something that we feel may hurt someone else’s feelings or cause someone else to be too upset, we may have to take your post or comment down. If we ever need to do this, we will send you a private message to explain why we did this. We encourage you to talk to us about it and let us know if you think it was the right thing to do.

10. Remember that if you leave your computer or device in a public space, other users could see what you post on the site. If you are leaving your device at any time, remember to log off of the site, your computer, or password protect your phone.
