# Supplementary material for: Role of Moderators on Engagement of Adolescents With Depression or Anxiety in a Social Media Intervention: Content Analysis of Web-Based Interactions
Source: JMIR Ment Health. 2019 Sep 26;6(9):e13467. doi: 10.2196/13467 (PMC6787534; doi:10.2196/13467)
Supplement: Multimedia Appendix 2 [file mental_v6i9e13467_app2.pdf]

Appendix 2: Blog post Codebook and Number Comments Coded

CODE NAME	DEFINITION	RESOURCE	A PRIORI NOTE	NUMBER COMMENTS CODED
**TYPE OF USER**				
**U: User**	User comment			96
**T: Team Member**	Team member (research assistant logging in as themselves not as a moderator) comment			28
**C: Community Member**	Community member (mental health advocate asked to join site) comment			6
**M: Moderator**	Moderator member comment			63
**SUPPORTIVE ACCOUNTABILITY CONCEPT**				
**Bond (M displays)**	The moderator appears emotionally attached/likes user.	Mohr Supportive Accountability Model (SA)	Would have to be something extraordinary because in general most comments are trying to convey this through appreciation	0
**Bond (UP)**	The user appears emotionally attached/likes moderator.	SA	Unlikely to find	0
**Trustworthy (M displays)**	The moderator displays trustworthiness	SA	Would have to be something extraordinary --- also in general moderators are trying to convey this	0
**Trustworthy (U perceives)**	The user perceives trustworthiness	SA	Unlikely to find --- maybe one quote where user trusts moderators' advice	0
**Benevolent (M perceives)**	The moderator displays benevolence	SA	Unlikely to find because not in	0

			context of a coaching relationship	
**Benevolent (U perceives)**	The user perceives benevolence	SA	Unlikely to find because not in context of a coaching relationship	0
**Expertise (MD)**	The moderator displays expertise	SA	Moderator shows that they have some expertise in behavioral health by the resources they provide or advice they give	22
**Expertise (UP)**	The user perceives expertise from the moderator	SA	Ok to code comments as expertise from the moderator when user is responding to a blogpost written by the moderator or a comment by the moderator	3
**Reciprocity (MD)**	The moderator displays that the user can benefit and the moderator can benefit from their relationship	SA	Unlikely to find	1
**Reciprocity (UP)**	The user perceives that the user can benefit and the moderator can benefit from their relationship	SA	Unlikely to find	0
**Definition (MD)**	The moderator involves user in the definition of their goals and values	SA		1
**Definition (UP)**	The user perceives they are being involved in the definition of their goals and values	SA		0

**Process Expectations (MD)**	Moderator focuses their expectations on processes (how) instead of outcomes (results)	SA	Unlikely to find because not a goal-setting intervention	0
**Process Expectations (UP)**	User perceives that the moderator is focusing their expectations on processes (how) instead of outcomes (results)	SA	Unlikely to find because not a goal-setting intervention	0
**U identifies problem**	User shows that they have identified a problem that they would like help solving	SA (Motivational)		14
**M offers problem help**	Moderator offers help for a problem the U has identified	SA (Motivational)		8
**U interested**	U feels site activities (reading/commenting) are engaging and interesting	SA (Motivational)	Any site activities count (not just interacting with the moderator)	87
**M increases interest**	M tries to increase U level of interest (help U see how online tasks might help in their life)	SA (Motivational)		21
**M verbally rewards**	M verbally rewards the U for their behavior (commenting) without seeking to control it or pressure	SA (Motivational)		45
**Mirroring**	U and M - or U and U - use same kind of emoticons or abbreviations or content, style, and tense of communications - this can lead to higher trust between each other (this is called entrainment)	SA (CMC - computer mediated communication)		1
**NOT Mirroring**	U and M - or U and U - DO NOT use same kind of emoticons or abbreviations or content, style, and tense of communications	SA (CMC)	Unlikely to find	0
**Not enough trust (MD)**	M displays they are not trustworthy	SA (CMC)	Unlikely to find	0
**Not enough trust (UP)**	U perceives the M is not trustworthy	SA (CMC)	Unlikely to find	0

**Not benevolence (MD)**	M displays they are unkind	SA (CMC)	Unlikely to find	0
**Not benevolence (UP)**	U perceives the M as unkind	SA (CMC)	Unlikely to find	0
**Low bond (MD)**	M displays having a low bond or less affection with the U	SA (CMC)	Unlikely to find	0
**Low bond (UP)**	U perceives they have a low bond or less affection with the M	SA (CMC)	Unlikely to find	0
**Absence of cues (MD)**	M is not sharing much about themselves (because in the absence of cues, the U has a positive impression)	SA (CMC)		0
**Offering cues (MD)**	M shares information about themselves	SA (CMC)		1
**SOCIAL SUPPORT CONCEPT**				
**Satisfaction with interaction**	Overall, how satisfied was the U with the **conversation** and the community's response	Kraut		14
**Seeking informational support**	U seeking informational support: messages provide information, advice, referrals or knowledge.	Kraut		4
**Providing informational support**	U or M providing informational support (information, advice, referrals, knowledge)	-- us --		96
**Providing emotional coping strategies**	U or M provided advice or demonstrate ways to successfully cope with difficult circumstances - will always be double coded with informational support	Kraut		37
**Seeking emotional support**	U seeking emotional support: trying to get understanding, encouragement, affirmation, sympathy, or caring.	Kraut		2
**Providing emotional support (ES)**	U or M providing emotional support (understanding, encouragement, affirmation, empathy, caring) -- can break down below			30

**ES: Providing non-specific emotional support**	U or M provides an emotionally supportive comment in general (not to a specific person)			17
**ES: Empathy--Down to their level**	U or M indicates that he or she understands what the recipient is going through and identifies with the recipient's emotional reactions and feelings, including painful and pleasurable feelings.	Kraut		4
**ES: Encouragement/ reassurance**	U or M offers encouragement express hope that situations will improve and/or support someone in their efforts when facing challenges.	Kraut		6
**ES: Appreciation**	U or M express appreciation for an individual and his or her accomplishments and provide support for someone's a sense of worth, value, and competence	Kraut		12
**ES: Caring--Virtual Hug**	U or M cares for or feels affection toward the recipient (overlap with encouragement)	Kraut		0
**ES: Expressions of concern**	U or M was concerned about the welfare of the recipient	Kraut		0
**Negative emotional self-disclosure**	U discusses negative thoughts or emotions, such as worry, sadness, or anger	Kraut		13
**Positive emotional self-disclosure**	U discusses positive thoughts or emotions, such as happiness, gratitude, and love.<br>--- what are they CURRENTLY disclosing about their emotional state	Kraut		29
**Neutral self-disclosure**	U discusses his or her experiences, events, or other facts about him or herself in a neutral, factual, and non-emotional way	Kraut		49

	--- what are they CURRENTLY disclosing about their emotional state			
**Value of intervention**	User expresses they found value in the intervention			47
**U gains new knowledge**				8
