# Supplementary material for: Role of Moderators on Engagement of Adolescents With Depression or Anxiety in a Social Media Intervention: Content Analysis of Web-Based Interactions
Source: JMIR Ment Health. 2019 Sep 26;6(9):e13467. doi: 10.2196/13467 (PMC6787534; doi:10.2196/13467)
Supplement: Multimedia Appendix 3 [file mental_v6i9e13467_app3.pdf]

Appendix 3: Moderator Interview Codebook

**Code name**	**Definition**
Role	Describing responsibilities and tasks of moderator
Most_Imp_Function	Most important function of the moderator
Least_Imp_Function	Least important function of the moderator
Training	Describing the training received for moderating
TrainingModifications	Opinion about how training could be improved
ComfortModerating	Describing how comfortable they felt with role of moderating
ModeratingChallenges	Describing what was challenging about the role of moderating
RoleModifications	Describing how the role of the moderator could be improved
EngagementChallenges	Describing challenges they had to engaging users to interact
Perceptions of Moderating	Description of their thoughts regarding the role of moderating (if they liked it or not)
Technical Challenges	Describing challenges with using website, Red Cap, and work phone.
