# Supplementary material for: Role of Moderators on Engagement of Adolescents With Depression or Anxiety in a Social Media Intervention: Content Analysis of Web-Based Interactions
Source: JMIR Ment Health. 2019 Sep 26;6(9):e13467. doi: 10.2196/13467 (PMC6787534; doi:10.2196/13467)
Supplement: Multimedia Appendix 4 [file mental_v6i9e13467_app4.pdf]

Appendix 4: Codes occurring more than 10 times in blog post comments

Code	Definition	Example comment
**Supportive accountability codes**		
Moderator (M) expertise	Moderator displays expertise.	*Remember you can ask almost anything! Successful therapy depends on personal disclosure and how well you connect with your therapist. Therefore, it's important to find a therapist you can trust and with whom you feel at ease.* (M)
User (U) identifies problem	U identifies a problem they would like help solving.	*I found the most peace in speaking to my grandma on my mother's side. Now that she passed, I find it more difficult to find peace with myself.* (U)
U interested	U feels site activities (reading or commenting) are engaging and interesting.	*This is great advice. Meeting with a therapist can be very uncomfortable. You need to be honest about painful and embarrassing things, and no one really wants to share those things, especially with a stranger.* (M)
M increases interest	M tries to increase U level of interest (help U see how web tasks might help in their life).	*Thanks for sharing! What are some ways you might try to word what you want to say from reading this article?* (M)
M verbally rewards	M verbally rewards the U for their behavior (commenting) without seeking	*You bring up a good point when talking about the importance of being able to trust your therapist. Ideally, a therapist is a person that you share things that you are uncomfortable sharing with anyone else. Trust is a key to building that rapport and the positive helpful relationship.* (M)

	to control it or pressure.	
**Social support codes**		
Satisfaction with interaction	Overall, how satisfied was the U with the conversation and the community's response.	*Thanks for starting to address this topic. I've met an increasing number of people who identify as pan-sexual. I was just discussing with a friend the difference between bisexual and pansexual. It's hard sometimes to wrap my small brain about these concepts. Just as I like the movement towards the gender nonbinary, I definitely like the move towards pan-sexuality.* (U)
Providing informational support	U or M providing informational support.	*Thank you for sharing. Losing someone close to us is very difficult. If you ever need to talk to someone, the crisis hotline is available 24/7 [at the crisis call center website]. Therapy is also helpful for grief, talking with a Mental Health provider about ways your Grandma was helpful, and you can continue to honor her memory by using some of those techniques with yourself or with others. I am sorry for your loss, thank you again for sharing.* (M)
Providing emotional coping strategies	U or M provided advice or demonstrate ways to successfully cope with difficult circumstances.	*This is so important to recognize in yourself, and consider for others. I didn't realize that my anxious thoughts were anxious until I shared them with a friend, because it is so common in my family history and my mom, though she worked so hard to be an awesome mother despite her depression and anxiety, modeled these unhealthy thoughts throughout my childhood. Now I can make the decision to think more logically, or if that is not working to use coping skills to work out my thoughts.* (U)

Providing emotional support (nonspecific)	U or M provides an emotionally supportive comment in general (not to a specific person).	*This is a great website for when you are studying and really could use a 2- or 5-minute chill break!* (U)
Providing emotional support (appreciation)	U or M expresses appreciation for an individual and his or her accomplishments.	*That is a really great point about feeling a sense of pride and accomplishment! It is something so simple but does make you feel industrious and like you have value because you have created something beautiful.* (M)
Negative emotional self-disclosure	U discusses negative thoughts or emotions, such as worry, sadness, or anger.	*I very rarely leave comments of any kind on any social platform out of fear I may insult or hurt someone.* (U)
Positive emotional self-disclosure	U discusses positive thoughts or emotions, such as happiness, gratitude, and love.	*This was so heartwarming and wonderful to see! Such a great way to end my day :slightly_smiling_face emoji:* (U)
Neutral self-disclosure	U discusses his or her experiences, events, or other facts about him or herself in a neutral, factual, and nonemotional way.	*When I am feeling stressed or am unable to fully concentrate on one thing, I tend to listen to music in order to better focus on the task at hand.* (U)

Value of intervention	User expresses they found value in the intervention.	*Emotional health is important. It's link to your physical health. I love this article. We should pay attention to our feelings and try to get a hold of them. Because we act based of how we are feelings. We all deserve peace. This article really hits home.* (U)
